# Unintended on-target chromosomal instability following CRISPR/Cas9 single gene targeting

**DOI:** 10.1016/j.annonc.2020.04.480

**Published:** 2020-09

**Authors:** J. Przewrocka, A. Rowan, R. Rosenthal, N. Kanu, C. Swanton

**Affiliations:** 1 The Francis Crick Institute, London, UK; 2 University College London, London, UK

Clustered regularly interspaced short palindromic repeats (CRISPR)/CRISPR-associated protein 9 (Cas9) seminal studies in mammalian cells^1^, ^2^, ^3^ have resulted in gene editing being broadly adopted in basic research. However, it has become apparent that the CRISPR/Cas9 system induces unintended off- and on-target genomic alterations^4^ and that there is a need for stricter clone screening methods before phenotypic characterisation is made, particularly before the technology is adopted for clinical purposes. Caution is also needed when working with cancer cell lines, as these often have underlying genomic instability and deficiencies in DNA repair or other safeguarding mechanisms which may permit large genomic deletions or rearrangements. Here, we report that CRISPR/Cas9 targeting of genes in close proximity to telomeres can result in chromosome arm truncations. We suggest assessing heterozygous single-nucleotide polymorphisms (SNPs) downstream of targeted genes to select clones without arm truncations. This screening approach could be applied alongside initial genotype assessment via sequencing at early stages of the experiment, prior to cell line expansion.

**Figure 1:**
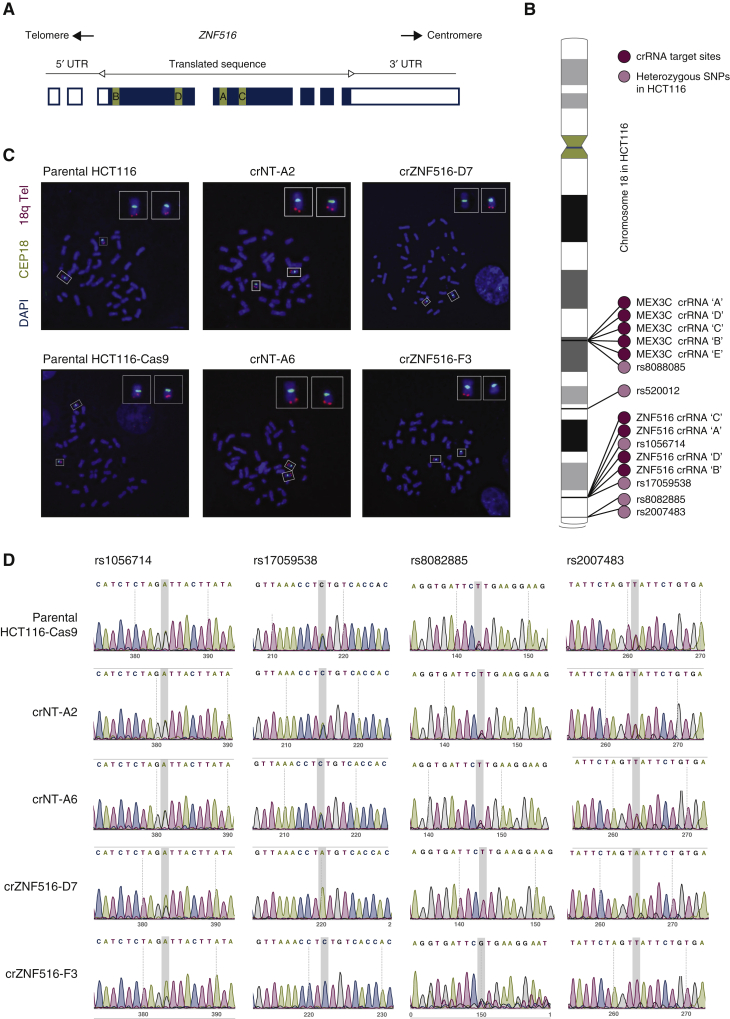
Detection of chromosome 18q arm truncation following CRISPR/Cas9 targeting of *ZNF516*. (A) Schematic of 7 exons of *ZNF516* on the reverse strand, with coding sequence shown as filled rectangles, and untranslated regions as empty rectangles. crRNA target sites are indicated in green in exon 3 and 4. (B) Schematic of heterozygous SNP location on chromosome 18 in HCT116. Schematic was made with Phenogram created by Ritchie Lab (2012). (C) FISH on metaphase spreads stained with DAPI (blue) and hybridised to a chromosome 18 centromere probe (green) and 18q subtelomeric region probe (red). (D) Sanger sequencing traces of four heterozygous SNPs in HCT116. rs1056714 is located in the *ZNF516* intron between crRNA-targeted exons 3 and 4. The rest of the SNPs are located distal to *ZNF516* and towards the telomere. crRNA, CRISPR (clustered regularly interspaced short palindromic repeats) RNA; DAPI, 4′,6-diamidino-2-phenylindole; SNP, single-nucleotide polymorphism; UTR, untranslated region.

We generated CRISPR/Cas9-mediated *ZNF516* knockout (KO) cell lines to characterise the role of *ZNF516* in colorectal cancer. We used an HCT116 cell line harbouring doxycycline-inducible Cas9 (HCT116-Cas9) to restrict temporal expression of the endonuclease and minimise off-target effects. Cells were transfected with either a pool of four CRISPR RNAs (crRNAs) against *ZNF516* (sites A–D in Figure 1A) or a pool of five nontargeting crRNAs, *trans*-activating crRNA, and treated with doxycycline for 5 days to induce Cas9 expression. After single-cell sorting, clones were expanded and screened for indel mutations using Sanger sequencing. ZNF516 protein levels were assessed by western blotting and messenger RNA expression levels assessed using quantitative PCR.

To undertake biological characterisation of *ZNF516* we performed RNA-seq in two *ZNF516* KO clones (crZNF516-D7 and crZNF516-F3) and two nontargeting clones (crNT-A2 and crNT-A6), to characterise gene expression changes upon *ZNF516* KO. Unexpectedly, we observed that a large proportion of the most significantly downregulated genes were located downstream of *ZNF516* towards the telomere of 18q. This observation was suggestive of a large-scale deletion or chromosome arm truncation following Cas9-induced double-strand break (DSB) in *ZNF516*.

To investigate the potential chromosome 18q arm truncation, we assessed loss of heterozygosity (LOH) of established heterozygous HCT116 SNPs, the location of which was obtained from the Sanger COSMIC Cell Line Project's genotyping calls (https://cancer.sanger.ac.uk/cell_lines/download). We examined four heterozygous SNPs, rs1056714 located in the *ZNF516* intron between targeted exons 3 and 4, and rs17059538, rs8082885 and rs2007483 distal to *ZNF516* (Figure 1B). Sanger sequencing revealed that both *ZNF516* KO clones had LOH of all SNPs downstream of *ZNF516*. Interestingly the *ZNF516* KO clones had lost a different allele, suggesting that it was equally possible to lose either of the chromosome arms. Furthermore, rs1056714 remained heterozygous, suggesting that arm truncation was due to a Cas9-induced DSB in exon 3 of *ZNF516*.

To further confirm arm truncation, we carried out FISH on metaphase spreads from parental cell lines, *ZNF516* KO and nontargeting clones, with chromosome 18 centromeric probes and 18q subtelomeric region probe. We detected subtelomeric probe signals on both copies of chromosome 18 in all cells (50 analysed) from parental cell lines and nontargeting clones, indicating the expected presence of 18q on both chromosomes 18 in HCT116 cells. By contrast, crZNF516 clones had detectable signal on only one chromosome 18 in all cells (50 analysed; Figure 1C). Since the homologous chromosomes 18 pairs have different morphologies in the HCT116 cell line, it was possible to confirm that different chromosomes were affected in the crZNF516-D7 and crZNF516-F3 clones (Figure 1C), confirming the result from Sanger sequencing.

To identify the prevalence of this phenomenon, we utilised next-generation sequencing and sequenced DNA fragments spanning ZNF516 crRNA target sites to assess the mutation rate, and fragments spanning heterozygous SNPs to assess chromosome 18q arm truncations in parallel. Of the 155 crZNF516 clones analysed (a sum of two independent experiments), 89 (57%) had indel mutations in at least one target site. Ten (6%) crZNF516 clones had LOH of all three SNPs below *ZNF516*, of which five also had LOH of the intronic SNP, suggesting arm truncation could occur due to DSB either in exon 3 or exon 4 (Table 1). Of the 107 crNT clones analysed (a sum of two independent experiments), none had indel mutations and one (1%) had LOH of all four SNPs analysed, suggesting that active Cas9 and/or transfection with nontargeting crRNAs could also induce large deletions.

**Table 1:** Prevalence of LOH of heterozygous SNPs following CRISPR/Cas9 targeting.

Cell line	Targeted gene	crRNA	Clones analysed	Clones with indels	Number of clones with indels at					Any SNP LOH	All ds^a^ SNP LOH
					Target site ‘A’	Target site ‘B’	Target site ‘C’	Target site ‘D’	Target site ‘E’		
HCT116	Nontargeting	Pool of 5 (Exp 1)	66	0	0	0	0	0	—	2 (3%)	0
HCT116	*ZNF516*	Pool of 4 (Exp 1)	96	65 (68%)	0	57 (59%)	21 (22%)	22 (23%)	—	35 (36%)	6 (6%)
HCT116	Nontargeting	Pool of 5 (Exp 2)	41	0	0	0	0	0	—	1 (2%)	1 (2%)
HCT116	*ZNF516*	Pool of 4 (Exp 2)	59	24 (40%)	20 (33%)	6 (10%)	1 (2%)	2 (3%)	—	6 (10%)	4 (7%)
HCT116	*ZNF516*	crRNA ‘A’	58	33 (57%)	33 (57%)	—	—	—	—	5 (9%)	4 (7%)
HCT116	*ZNF516*	crRNA ‘B’	48	17 (35%)	—	17 (35%)	—	—	—	2 (4%)	1 (2%)
HCT116	*ZNF516*	crRNA ‘C’	47	4 (9%)	—	—	4 (9%)	—	—	3 (6%)	1 (2%)
HCT116	*ZNF516*	crRNA ‘D’	53	13 (25%)	—	—	—	13 (25%)	—	3 (6%)	2 (4%)
HCT116	Nontargeting	Pool of 5	58	0	0	0	0	0	0	1 (2%)	0
HCT116	*MEX3C*	Pool of 5	52	15 (29%)	0	5 (10%)	2 (4%)	10 (19%)	1 (2%)	0	0

crRNA, CRISPR (clustered regularly interspaced short palindromic repeats) RNA; LOH, loss of heterozygosity; SNP, single-nucleotide polymorphism.

a ds, downstream from the targeted gene towards the telomere.

In parallel, we transfected HCT116-Cas9 cells with an individual crRNA (‘A’, ‘B’, ‘C’ or ‘D’; target sites shown in Figure 1A) to investigate whether this would have the same effect as the pool of four crRNAs. Depending on the crRNA used, mutation rates varied from 9% for crRNA ‘C’ to 57% for crRNA ‘A’ (Table 1). The proportion of samples affected by LOH of all heterozygous SNPs downstream of *ZNF516* varied between 2% for crRNA ‘C’ and 7% for crRNA ‘A’, indicating that perhaps the efficiency of a particular crRNA to induce mutation could correlate with its efficiency of chromosome arm truncation. Results for all crRNA target sites are summarised in Table 1.

Finally, we targeted *MEX3C* located further away from the telomere than *ZNF516*, with a pool of five crRNAs (Figure 1B) to investigate whether proximity to the telomere influences arm truncation prevalence. Of the 52 crMEX3C clones analysed, 15 (29%) had indel mutations and none had LOH of the two SNPs analysed below *MEX3C* (rs8088085 and rs520012; Figure 1B; Table 1). Of the 58 crNT clones analysed, none had indel mutations and one (2%) had LOH of rs8088085, again suggesting that active Cas9 and/or transfection with nontargeting crRNAs could also induce large deletions.

In this study we demonstrate that CRISPR/Cas9 targeting can induce inadvertent arm truncation. While the existence of a low-background LOH of 18q in parental HCT116-Cas9 cells is a possibility even without CRISPR/Cas9 intervention, it seems that targeting genes with close proximity to telomeres could elevate the extent of chromosomal arm deletions compared with a commonly used nontargeting control.

Two studies^5^^,^^6^ previously reported incidental chromosome arm truncation following CRISPR/Cas9 targeting telomere-proximal genes: *POLE*^5^ located 11 kb and *UROS*^6^ located 6 million bp away from chromosome arm end. *ZNF516* is located 6 million bp from the telomere, whereas *MEX3C*, targeting which did not induce arm truncation, is located 31 million bp away from the arm end. This could suggest that targeting genes close to telomeres could result in arm truncations. Reports by Rayner et al.^5^ and Cullot et al.^6^ suggested detection of arm truncation with FISH analysis for targeted genes; however, this can only be achieved after cell colony expansion and involves several experimental steps. We suggest assessing heterozygous SNPs downstream of targeted genes in addition to initial mutation analysis and genotype confirmation via sequencing. This methodology could preselect clones with correct genotype and without arm truncations before further cell line expansion for future downstream characterisation.
